# Metabolic Syndrome and Rheumatoid Arthritis Activity: An Analysis of Clinical, Laboratory, and Ultrasound Parameters

**DOI:** 10.3390/nu15224756

**Published:** 2023-11-12

**Authors:** Krzysztof Grzechnik, Bożena Targońska-Stępniak

**Affiliations:** 1 Department of Rheumatology and Connective Tissue Diseases, Independent Public Teaching Hospital No. 4, Jaczewskiego 8, 20-059 Lublin, Poland; krzysiek.grzechnik@gmail.com; 2 Department of Rheumatology and Connective Tissue Diseases, Medical University of Lublin, Jaczewskiego 8, 20-059 Lublin, Poland

**Keywords:** rheumatoid arthritis, metabolic syndrome, ultrasound examination

## Abstract

(1) Background: Rheumatoid arthritis (RA) is a chronic autoimmune disease associated with an increased incidence of metabolic syndrome (MetS). The aim of this study was to determine if there is an association between MetS and parameters of RA activity, as well as between metabolic parameters and indices of RA activity. (2) Methods: This study involved 65 patients with RA. MetS was diagnosed according to the 2009 IDF/AHA/NHLBI criteria. The comparative analysis was conducted between RA patients with MetS (RA (MetS (+)) and without MetS (RA (MetS (−)). The activity of RA was assessed using clinical, laboratory, and ultrasound (US) parameters. (3) Results: Compared with RA MetS (−) patients, RA MetS (+) patients were characterized by higher disease activity, according to Disease Activity Score (DAS28), Simplified Disease Activity Index (SDAI), and Clinical Disease Activity Index (CDAI). RA MetS (+) patients had significantly higher tender and swollen joint counts, and values of erythrocyte sedimentation rate, *C*-reactive protein, and US parameters (grey-scale (GSUS), power Doppler (PDUS)). Significant correlations were found between metabolic parameters (waist circumference, cholesterol and glucose concentrations) and indices of RA activity. (4) Conclusion: The results of this study show that, in patients with RA, the presence of MetS is associated with higher disease activity, based on several clinical, laboratory, and US parameters.

## 1. Introduction

Rheumatoid arthritis (RA) is a chronic autoimmune disease characterized by symmetric arthritis leading to irreversible joint damage [1]. The active form of RA may be associated with internal organs involvement, metabolic disorders, and premature atherosclerosis. Nowadays, RA is considered an independent risk factor for cardiovascular diseases (CVDs) [2]. The risk of CVD in patients with RA in comparison with the general population is increased by approximately 50%, and CVD is regarded as the main cause of death [3]. The incidence of traditional CVD risk factors (central obesity, hypertension, dyslipidemia, insulin resistance) is high in patients with RA and might occur as metabolic syndrome (MetS) [2,3].

MetS is defined as a cluster of interrelated metabolic factors which increase the risk of CVD, atherosclerosis, and type 2 diabetes [2,4]. It is reported that in patients with MetS, CVDs occur more often, and the risk of complications is greater. In addition, the more components of MetS that are present, the higher the cardiovascular (CV) mortality rate [2].

The high incidence of MetS in patients with RA and the significant relationship between MetS and RA were reported in several studies [2,5,6,7]. MetS was found in 16.2% to 40.9% of women with RA, depending on the criteria for MetS, and was higher than in controls (10.5% to 22.9%) [2]. According to a large meta-analysis, the overall prevalence of MetS in patients with RA was 30.7%, and varied from 14.3% to 37.8%, depending on the criteria used. In Europe, the prevalence of MetS was estimated at 35.2% based on the International Diabetes Federation (IDF) criteria [7]. Individual components of MetS (e.g., hypertension, dyslipidemia, abdominal obesity) were found more often in patients with RA than in controls [2].

Central (abdominal) obesity and insulin resistance seem to be the key factors in the development of MetS. Significant relationships have been reported between central obesity and individual components of MetS. Obesity is associated with chronic, low-grade inflammation [2]. Higher levels of interleukin 6 (IL-6) and tumor necrosis factor (TNF) α were found in patients with several features of MetS such as central obesity and insulin resistance, when compared to the general population [5,8]. The overproduction of pro-inflammatory adipokines (e.g., leptin, resistin, visfatin) is observed as a result of hypoxia and local inflammation in the hypertrophic adipose tissue. These adipokines induce neutrophil chemotaxis, activate monocytes and macrophages enhancing their phagocytic activity, and stimulate the pro-inflammatory activity of lymphocytes [9]. Specific body composition changes may be found in patients with active RA, defined as rheumatoid cachexia (RC), and may affect approximately 20% of patients with RA [10]. The total body weight usually remains stable in RC, despite the reduced lean body mass, which is compensated by an increased fat mass. The excess visceral body fat in RA patients was reported to be associated with an increased risk of hypertension, insulin resistance, and MetS [2,11]. It seems that RC may accelerate morbidity and mortality in patients with RA and is associated with MetS [2].

The lipid paradox is observed in patients with active RA, with an inverse relationship between excessive inflammation and cholesterol levels. The mechanisms may include an impairment of cholesterol turnover due to high levels of acute phase reactants; increased uptake of low-density lipoprotein (LDL) and oxidized LDL by liver cells induced by *C*-reactive protein (CRP) [2]. In active RA, the lipid paradox related to high-grade inflammation is associated with accelerated atherosclerosis progression and a higher CVD risk.

Reports in the literature indicate that the presence of MetS in RA patients was associated with a higher erythrocyte sedimentation rate (ESR), disease activity score, health assessment score, swelling of large joints, and lower methotrexate (MTX) dose [2]. However, there are a few studies in the literature reporting a relationship between MetS and RA activity. 

The activity of RA can be evaluated by different parameters: clinical, laboratory, and ultrasound (US). In the last decade, an increasing impact of US usage in rheumatology has been observed [12]. US has become a fundamental tool in the work of a rheumatologist, providing a valuable complement to clinical examinations. US examination is much more sensitive in detecting synovitis compared to clinical assessments [13]. To the best of our knowledge, this is the first study assessing the relationship between MetS and RA activity that includes US assessments.

The aim of this study was to determine associations between MetS and RA activity which was assessed with several parameters (clinical, laboratory and US), as well as the relationships between metabolic parameters and indices of RA activity.

## 2. Materials and Methods

### 2.1. The Study Population

All the patients included in the study were treated at the Department of Rheumatology and Connective Tissue Diseases of the Medical University of Lublin, Poland. The study was conducted in accordance with the Declaration of Helsinki, and was approved by the Ethics Committee of the Medical University of Lublin (approval number KE-0254/214/2020). Written informed consent was obtained from each patient prior to enrollment in the study.

The study population consisted of 65 patients, who were over 18 years old, with a diagnosis of RA according to the 2010 ACR/EULAR (American College of Rheumatology/European Alliance of Associations for Rheumatology) criteria [1]. The exclusion criteria was current treatment with biological or targeted synthetic disease-modifying anti-rheumatic drugs (DMARDs). 

All RA patients were diagnosed for MetS according to the IDF/AHA/NHLBI (International Diabetes Federation/American Heart Association/National Heart, Lung and Blood Institute) 2009 criteria [5]. 

The patients were divided into two groups: 23 patients with RA who met the criteria for MetS (RA MetS (+)), and 42 patients who did not meet the criteria for MetS (RA MetS (−)). The comparative analyses were conducted between these two groups of patients.

### 2.2. Clinical and Laboratory Assessment

Demographic and clinical data were obtained through medical interviews and reviews of the medical history. The physical examination included measurements of body weight, height, and waist circumference, and a peripheral joint assessment. Body mass index (BMI) was calculated as body weight/height^2^ (kg/m^2^). 

The blood samples for the laboratory tests were collected in the morning, after overnight fasting. The testing measured complete blood cell count, CRP, ESR, triglycerides (TG), total cholesterol (TC), LDL-C, high-density lipoprotein cholesterol (HDL-C), and glucose levels. The samples were analyzed with an ADVIA 2120i System automated cell counter (Siemens, Munich, Germany).

### 2.3. Ultrasound (US) Assessment

Each patient underwent a US examination of 22 small joints of the hands (both wrist joints, 10 metacarpophalangeal joints, 8 proximal interphalangeal joints, and both thumb interphalangeal joints) with an assessment of the degree of synovial hypertrophy (grey-scale US, GSUS) and vascularization (Power Doppler US, PDUS) based on the four-level semi-quantitative EULAR-OMERACT scale [14]. According to the EULAR recommendations, joints were assessed in the longitudinal projection, using, respectively, dorsal (wrist) and dorsal and palmar (other joint) views [15]. The highest score from the dorsal or palmar view was used for statistical calculations. The cumulative degree of synovial hypertrophy in GSUS and the cumulative degree of synovial vascularization in PDUS were obtained by summing the scores acquired in the assessment of individual joints (range 0–66). In all patients, the ultrasound examination was performed by the same rheumatologist using the same machine (MyLab25 Gold, Esaote, Genova, Italy) equipped with a 4 cm linear transducer with an imaging frequency of 18 MHz. For the PD option, the pulse repetition frequency was set to 1 kHz and the gain just below the level generating random noise.

### 2.4. Assessment of RA Activity

The activity of RA was evaluated by clinical, laboratory, and US parameters. The clinical parameters included tender joint count (TJC), swollen joint count (SJC), duration of morning stiffness, and Visual Analogue Scale (VAS) for disease activity according to the patient (PGA) and physician (PhGA). The ability to perform daily activities was assessed using the modified Health Assessment Questionnaire (M-HAQ), with scores ranging from 0 to 3 (score of 0 represents no impairment of function) [16]. The laboratory parameters included ESR and CRP. The US parameters included semiquantitative assessments of GSUS and PDUS. The activity of RA was calculated using three indices: the Disease Activity Score in 28 joints (DAS 28) [17], Clinical Disease Activity Index (CDAI) [18], and Simplified Disease Activity Index (SDAI) [19].

### 2.5. Statistical Analysis

The Kolmogorov–Smirnov test was used to assess the normality of the data distribution. Parametric variables are presented as the mean ± standard deviation (SD) and non-parametric variables are presented as the median and interquartile range (IQR). Categorical data are presented as numbers and percentages. The Student’s *t*-test (for parametric data) and Mann–Whitney U test (for non-parametric data) were used to compare continuous variables in subgroups. Categorical data were compared using the chi-square test. Correlation between quantitative variables was assessed using the Pearson or Spearman correlation test for parametric and non-parametric variables, respectively. When several correlations were conducted simultaneously, the Bonferroni correction was applied. For variables that showed statistically significant correlations, an additional multiple linear regression analysis was conducted. For all tests, *p* values < 0.05 were considered significant. The calculations were performed using the Statistica 14.0.0.1 application.

## 3. Results

### 3.1. Characteristics of the Study Group 

The detailed characteristics of the study group are presented in Table 1. The study group consisted mainly of women (87.7%). The mean age of the patients was approximately 50 years, and the mean duration of the disease was about 12 years. The vast majority of patients were positive for antibodies: 80% had anti-citrullinated peptide antibodies (ACPAs) and 87.7% had anti-rheumatoid factor IgM (RF-IgM). Approximately 40% of the patients reported being smokers (Table 1). At the time of examination, 55 patients were treated with conventional synthetic DMARDs, most commonly with MTX (dose 10–25 mg/week, in monotherapy or combination). Glucocorticosteroids (GCs) were used in 44 patients (prednisone dose: 5–10 mg/day) (Table 1).

MetS was confirmed in 23 (35.4%) RA patients and more often in male (62.5%) than in female (31.6%) patients (not statistically significant) (Table 1). Compared with RA MetS (−) patients, RA MetS (+) patients had a significantly larger waist circumference, and higher BMI, TG, and glucose levels, as well as a lower HDL-C concentration (Table 1).

There were no significant differences between RA MetS (+) and RA MetS (−) patients with regard to age, gender, disease duration, positivity for ACPA and RF-IgM, as well as DMARD and GCS use (Table 1).

### 3.2. Clinical Parameters of RA Activity in RA MetS (+) vs. RA MetS (−) Patients

RA MetS (+) patients were characterized by significantly higher disease activity as assessed by the three indices (DAS28, SDAI, CDAI) compared to RA MetS (−) patients (Table 2). 

RA MetS (+) patients also had significantly higher TJC and SJC, as well as a longer duration of morning stiffness (Table 2).

No significant differences were found with regard to PGA, PhGA, and M-HAQ values (Table 2).

### 3.3. Laboratory Parameters of RA Activity in RA MetS (+) vs. RA MetS (−) Patients

**Figure 1:**
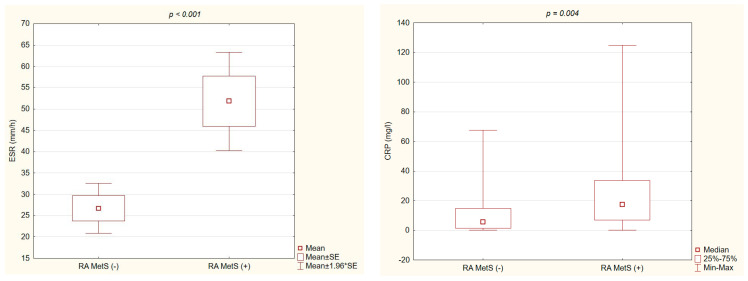
Significant differences of ESR value and CRP concentration between the groups of RA MetS (+) vs. RA MetS (−) patients.

Significantly higher values of ESR and CRP were found in RA MetS (+) patients compared with RA MetS (−) patients (Figure 1).

### 3.4. Ultrasound Parameters of RA Activity in RA MetS (+) vs. RA MetS (−) Patients

**Figure 2:**
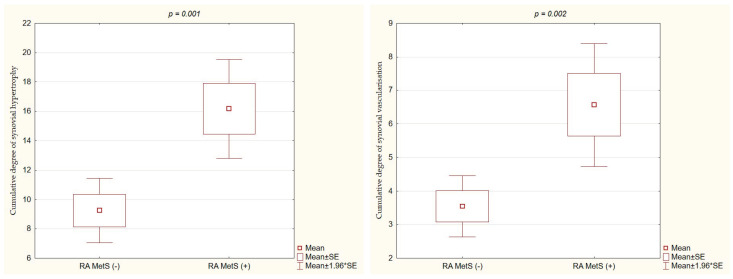
Significant differences of cumulative degree of synovial hypertrophy in GSUS and cumulative degree of synovial vascularization in PDUS, between the groups of RA MetS (+) vs. RA MetS (−) patients.

Compared with RA MetS (−) patients, RA MetS (+) patients had significantly higher values of cumulative degree of synovial hypertrophy in GSUS (16.2 ± 8.2 vs. 9.2 ± 7.3, *p* = 0.001) (Figure 2) and cumulative degree of synovial vascularization in PDUS (6.6 ± 4.5 vs. 3.6 ± 3, *p* = 0.002) (Figure 2).

### 3.5. Correlations between Metabolic and RA Activity Parameters

Significant positive correlations were found between BMI and ESR, as well as between waist circumference and M-HAQ, DAS 28, and inflammatory parameters (Table 3). Significant negative correlations were found between TC level and SJC, SDAI, and CDAI, as well as between HDL-C level and duration of morning stiffness, DAS28 score, SDAI, CDAI, and inflammatory parameters (CRP, ESR) (Table 3). Significant positive correlations were also observed between glucose level and TJC, duration of morning stiffness, and M-HAQ score (Table 3).

In the multiple linear regression analysis, statistically significant positive correlations were confirmed between waist circumference and M-HAQ score, DAS28 score, and ESR (Table 4). Significant negative correlations were found between TC level and SDAI, as well as for HDL-C level with duration of morning stiffness, DAS28 score, and ESR (Table 4). A significant positive correlation was confirmed between glucose level and M-HAQ score (Table 4).

## 4. Discussion

The results of this study demonstrated the relationship between MetS and disease activity in patients with RA. The presence of MetS in RA patients was associated with significantly higher disease activity, as assessed by clinical, laboratory, and US parameters. In patients with MetS, we observed a higher TJC, SJC, and duration of morning stiffness, as well as higher values for three indices (DAS28, SDAI, CDAI). Higher values of laboratory parameters (CRP, ESR) were found in patients with MetS compared to those without MetS. The US examination was performed to provide additional parameters for RA activity evaluation. The results confirmed a higher degree of both synovial hypertrophy (GSUS) and synovial vascularization (PDUS) in patients with MetS, which points to higher activity of inflammatory processes within the synovial tissue of affected joints. This was the first study reporting the relationship between MetS and RA activity that was assessed using US parameters.

In this study, MetS was observed in over 1/3 of the patients with RA, which is consistent with the data in the literature [6,20]. Only a few studies in the literature examined the impact of MetS on RA activity, and the results were inconsistent. A higher SJC was reported in patients with MetS [21] while another study conducted in 100 patients with newly diagnosed RA and 100 patients already being treated for RA did not find a relationship between TJC and SJC and the presence of MetS [22]. 

Our results demonstrated a relationship between MetS and a significantly longer duration of morning stiffness in RA patients. This association had not been previously described in the literature. Morning stiffness significantly affects the quality of life of patients, greatly impeding their ability to carry out daily activities, and is mentioned as a reason for early termination of professional activity by RA patients [23]. Considering the impact of morning stiffness on the patient’s quality of life, this observation seems to be important.

This study revealed a significant association between the presence of MetS and higher RA activity, as determined by three indices: DAS28, SDAI, and CDAI. Most studies in the literature evaluated an association between MetS and RA activity using DAS28 only. Our findings are in agreement with the reports in the literature [22,24,25], which indicate that RA patients with MetS, compared to those without MetS, had significantly higher DAS28 scores. However, other studies showed no relationship between MetS and DAS28 [21,26,27,28]. There is only one study, besides this one, which evaluated RA activity in patients with and without MetS using three indices (DAS28, SDAI, and CDAI). Notably, the results of that analysis align with our findings [29].

In our study, the ability to perform daily activities according to the M-HAQ was not significantly different in patients with and without MetS, which is consistent with prior studies [22,27,30]. Other studies showed a significant relationship between the presence of MetS in RA patients and a higher M-HAQ score [31,32].

The results of this study confirmed that RA MetS (+) patients had significantly higher inflammatory parameters (ESR, CRP) compared to MetS (−) patients, despite comparable ages, disease durations, and treatments. Most studies in the literature support the finding that RA patients with MetS, compared to those without MetS, have significantly higher ESR values [29,32,33,34]. The CRP concentration was reported to be higher in patients with MetS [28,33]. However, in other studies, no significant relationship between ESR, CRP levels, and the occurrence of MetS in RA patients was found [27,32]. The higher activity of RA in patients with MetS was confirmed in our study by US examination, along with a significantly higher cumulative degree of synovial hypertrophy in GSUS and synovial vascularization in PDUS. The consistency between the US assessment of RA activity, and the clinical and laboratory parameters of disease activity has been reported in the literature [13]. 

In this study, significant correlations were found between metabolic and RA activity parameters. We observed that waist circumference, and serum glucose, TC, and HDL-C levels exhibited significant correlations with RA activity parameters. Waist circumference and glucose concentration correlated positively with M-HAQ score, suggesting an impairment in daily activity and reduced quality of life. It is worth emphasizing that these parameters (excluding TC level) are included in the criteria of MetS.

There were positive correlations between waist circumference and DAS28 score, ESR, and M-HAQ score, which is consistent with the data in the literature [21,35]. In contrast, in other studies, no correlations with waist circumference were observed [36,37]. In this study, a negative correlation was found between serum TC level and SDAI, as well as a negative correlation between serum HDL-C level and duration of morning stiffness, DAS28 score, and ESR. In the literature, a negative correlation was observed between serum TC level and SJC, and CRP level [38] or no significant correlation was found between TC level and RA activity parameters [21]. In other studies, a negative correlation was observed between HDL-C and CRP levels and ESR, but not with clinical indicators of RA activity [39] or there was a negative correlation between HDL-C level and DAS28 score [40]. However, in other studies, no significant correlations were found between HDL-C level and DAS28 score, and inflammatory parameters [21,37]. DMARD treatment is crucial to inhibit the activity of the disease and, as a consequence, may decrease the risk of metabolic abnormalities associated with inflammation. It is reported that methotrexate is associated with a reduced risk of MetS development [2]. Anti-TNF drugs may regulate the lipoprotein spectrum, and they seem to reduce CVD risk in RA patients [3]. Tocilizumab was reported to improve the insulin resistance in RA patients [3]. Currently, there are other novel types of treatment considered for patients with RA, including probiotics [41] and nanotherapies [42].

Our findings indicate that RA patients should undergo diagnostic procedures for MetS. A diagnosis of MetS will enable the implementation of therapeutic management, which may reduce metabolic disorders and therefore reduce RA activity, increasing the chance for remission and improvement the patient’s quality of life.

This study has limitations. One of them is the small size of the study group. The second is the recruitment of patients from a hospital facility, which does not allow for a cross-sectional assessment of the entire population of RA patients. A third limitations is that we did not assess the impact of MetS on treatment response and the effect of weight reduction on RA activity. Therefore, a further prospective studies involving a large group of patients and additional parameters seem to be necessary. Another limitation is that multivariate analysis would have been needed, but was not possible due to the small group sizes.

The study has several strengths. To the best of our knowledge, this is the first study assessing the relationship between MetS and US assessments of RA activity. Another strength is the assessment of RA activity through clinical, laboratory, and US parameters. In addition, in contrast to most publications in the literature which only evaluated individual parameters, this study analyzed multiple parameters to assess the relationship between MetS and RA activity.

## 5. Conclusions

The results of this study confirmed significant associations between MetS and RA activity. Compared with RA patients without MetS, those with MetS had higher disease activity, as assessed with several parameters (clinical, laboratory, and US). Significant correlations were found between metabolic parameters (waist circumference, and cholesterol and glucose concentrations) and indices of RA activity. This was the first study to demonstrate an association between MetS and US parameters of RA activity.

## Figures and Tables

**Table 1:** Demographic and clinical characteristics of RA MetS (+) and MetS (−) patients.

	All RA Patients	RA MetS (+)	RA MetS (−)	*p* Value MetS (+) vs. MetS (−)
Female/Male (n, %)	57 (87.7)	18 (78.3)	39 (92.9)	NS
	8 (12.3)	5 (21.7)	3 (7.1)	
Age (years)	50.1 (±16.2)	53.91 (±15.5)	48.07 (±16.4)	NS
Height (cm)	165.9 (±8)	167.22 (±8.1)	165.11 (±8)	NS
Waist circumference (cm)	88.3 (±11.2)	95.5 (±7.8)	84.4 (±10.9)	<0.001
BMI (kg/m^2^)	24.8 (±3.8)	26.8 (±3.5)	23.7 (±3.6)	0.001
TC (mg/dl)	188.9 (±35)	189.4 (±42.9)	188.7 (±30.4)	NS
HDL-C (mg/dl)	57.6 (±14.4)	50.3 (±11.7)	61.7 (±14.2)	0.002
LDL-C (mg/dl)	109.6 (±29.9)	113.8 (±34.4)	107.4 (±27.2)	NS
TG (mg/dl)	106.1 (±44.1)	121.1 (±53.5)	98 (±36.1)	0.042
Glucose (mg/dl)	92.5 (±16.9)	100.8 (±23)	88.1 (±10.3)	0.004
Disease duration (months)	147.2 (±115.8)	169.17 (±128.9)	135.19 (±107.7)	NS
ACPA (n, %)	52 (80)	21 (91.3)	31 (73.8)	NS
RF-IgM (n, %)	57 (87.7)	22 (95.7)	35 (83.3)	NS
Current GCS treatment (n, %)	44 (67.7)	16 (69.6)	28 (66.7)	NS
Current MTX treatment (n, %)	43 (66.2)	16 (69.6)	27 (64.3)	NS
Current SS treatment (n, %)	4 (6.2)	1 (4.4)	3 (7.1)	NS
Current LEF treatment (n, %)	5 (7.7)	0 (0.0)	5 (11.9)	NS
Current antimalarial drugs treatment (n, %)	24 (36.9)	11 (47.8)	13 (31)	NS

Values are displayed as mean ± standard deviation (SD) or frequencies with corresponding percentages (%). ACPA, anti-citrullinated peptide antibodies; BMI, body mass index; GC, glucocorticoid; HDL-C, high-density lipoprotein cholesterol; LDL-C, low-density lipoprotein cholesterol; LEF, leflunomide; MTX, methotrexate; RF-IgM, rheumatoid factor IgM; SS, sulfasalazine; TC, total cholesterol; TG, triglyceride.

**Table 2:** Comparison of clinical parameters of RA activity in RA MetS (+) vs. RA MetS (−) patients.

	RA MetS (+) (n = 23)	RA MetS (−) (n = 42)	*p* Value
DAS28	5.62 (±1.2)	4.53 (±1.4)	0.002
CDAI	25.54 (±11.3)	18.85 (±8.8)	0.01
SDAI	28.65 (±12.9)	20.43 (±9.8)	0.005
TJC	9.4 (±5.5)	5.8 (±3.8)	0.003
SJC	6.5 (±3.8)	4.0 (±2.9)	0.003
Morning stiffness (minutes)	80 (30–120)	50 (15–60)	0.017
PGA (VAS) (mm)	50.3 (±24.9)	51.05 (±2.3)	NS
PhGA (VAS) (mm)	46.0 (±17)	39.6 (±17.9)	NS
M-HAQ	1.4 (±0.6)	1.1 (±0.7)	NS

Values are displayed as mean ± standard deviation (SD) or median (IQR). CDAI, Clinical Disease Activity Index; DAS28, Disease Activity Score in 28 joints; M-HAQ, Modified Health Assessment Questionnaire; PGA, patient global assessment; PhGA, physician global assessment; SDAI, Simplified Disease Activity Index; SJC, swollen joint count; TJC, tender joint count; VAS, Visual Analogue Scale.

**Table 3:** Correlations between metabolic and RA activity parameters.

	BMI	Waist Circumference	TC	HDL-C	LDL-C	TG	Glucose
**TJC**	NS	NS	NS	NS	NS	NS	r = 0.33
							*p* = 0.009
**SJC**	NS	NS	r = −0.3	NS	NS	NS	NS
			*p* = 0.014				
**Morning stiffness**	NS	NS	NS	rs = −0.38	NS	NS	rs = 0.33
				*p* = 0.002			*p* = 0.008
**M-HAQ**	NS	r = 0.36	NS	NS	NS	NS	r = 0.35
		*p* = 0.003					*p* = 0.005
**DAS28**	NS	r = 0.36	NS	r = −0.35	NS	NS	NS
		*p* = 0.004		*p* = 0.004			
**SDAI**	NS	NS	r = −0.33	r = −0.31	NS	NS	NS
			*p* = 0.008	*p* = 0.012			
**CDAI**	NS	NS	r = −0.3	r = −0.28	NS	NS	NS
			*p* = 0.01	*p* = 0.02			
**CRP**	NS	r = 0.34	NS	rs = −0.32	NS	NS	NS
		*p* = 0.005		*p* = 0.01			
**ESR**	r = 0.3	r = 0.45	NS	r = −0.34	NS	NS	NS
	*p* = 0.015	*p* < 0.001		*p* = 0.006			

BMI, body mass index; CDAI, Clinical Disease Activity Index; CRP, *C*-reactive protein; DAS28, Disease Activity Score in 28 joints; ESR, erythrocyte sedimentation rate; HDL-C, high-density lipoprotein cholesterol; LDL-C, low-density lipoprotein cholesterol; M-HAQ, Modified Health Assessment Questionnaire; SDAI, Simplified Disease Activity Index; SJC, swollen joint count; TC, total cholesterol; TG, triglyceride; TJC, tender joint count.

**Table 4:** Multiple linear regression analysis for correlations between metabolic parameters and RA activity indices.

	Waist Circumference	TC	HDL-C	Glucose
**Morning Stiffness**	NS	NS	b = −1.14	NS
			*p* = 0.01	
**M-HAQ**	b = 0.02	NS	NS	b = 0.01
	*p* = 0.02			*p* = 0.02
**DAS28**	b = 0.39	NS	b = −0.3	NS
	*p* = 0.007		*p* = 0.007	
**SDAI**	NS	b = −0.08	NS	NS
		*p* = 0.04		
**ESR**	b = 1.56	NS	b = −0.58	NS
	*p* < 0.001		*p* = 0.005	

DAS28, Disease Activity Score in 28 joints; ESR, erythrocyte sedimentation rate; HDL-C, high-density lipoprotein cholesterol; M-HAQ, Modified Health Assessment Questionnaire; SDAI, Simplified Disease Activity Index; TC, total cholesterol.

## Data Availability

All data reported in this study are available upon request by contacting the corresponding author.
